# Partially hydrolyzed cow’s milk protein formula with an added prebiotic is well-tolerated, safe, and supports age-appropriate growth in healthy term infants through one year of age: DBRCT

**DOI:** 10.1186/s12887-025-06454-2

**Published:** 2026-01-13

**Authors:** Veronica Fabrizio, Salma A. Abdelmagid, Ashley Bose, Max Hale, E Carlton Hays, Michael Hudson, Teena Hughes, Daniel Leonard, Kevin Rouse, Maria Sideri, Jami Walker, Jennifer L. Wampler, Michael Yeiser, Weihong Zhuang, Steven S. Wu

**Affiliations:** 1 Evidence Generation and Clinical Research, Mead Johnson Nutrition, Evansville, IN USA; 2 Regulatory Affairs, Mead Johnson Nutrition, Toronto, ON Canada; 3 PAS Research, McAllen, TX USA; 4 Birmingham Pediatric Associates, Birmingham, AL USA; 5 The Jackson Clinic – North Jackson, Jackson, TN USA; 6 DCOL Center for Clinical Research, Longview, TX USA; 7 PAS Research, Tampa, FL USA; 8 Meridian Clinical Research, Hastings, NE USA; 9 The Children’s Clinic of Jonesboro, PA, Jonesboro, AR USA; 10 Nutrition Science, Mead Johnson Nutrition, Amsterdam, The Netherlands; 11 Spring Medical Research, Owensboro, KY USA; 12 Indiana University School of Medicine, Evansville, IN USA

**Keywords:** Infant formula, Partially hydrolyzed cow’s milk protein, Prebiotics, Polydextrose, Galactooligosaccharides, Growth

## Abstract

**Background:**

Partially hydrolyzed cow’s milk protein (PHP) formulas are nutritionally complete and have a high-quality protein composition, and extensive history of safe use. The current study evaluated growth and safety in healthy term infants receiving a new PHP formula with an added prebiotic blend.

**Methods:**

In this multi-center, double-blind, controlled, parallel, prospective study, healthy term infants were randomized to receive one of two formulas through 365 days of age: previously marketed intact cow’s milk protein formula (Control, *n* = 122) or investigational PHP formula (INV-PHP, *n* = 122). Both formulas had an added prebiotic blend of polydextrose (PDX) and galactooligosaccharides (GOS) (1:1, 4 g/L). The primary outcome was rate of weight gain (g/day) from 14 to 120 days of age. To establish equivalence between study formulas, the 90% two-sided confidence interval (CI) of the mean group difference in body weight growth rate from 14 to 120 days of age needed to be contained within a predefined equivalence interval (± 3 g/day). Growth rates through Day 120 and achieved anthropometrics through Day 365 were analyzed by ANOVA. Parent-reported tolerance outcomes were also collected. Medically confirmed adverse events were collected throughout the study period.

**Results:**

Of 244 infants enrolled and randomized (Control, *n* = 122; INV-PHP, *n* = 122); 175 completed study feeding through Day 120 (Control, *n* = 91; INV-PHP, *n* = 84). Equivalence in rate of weight gain from 14 to 120 days of age was demonstrated with the difference in means of 0.5 g/day and 90% CI [− 1.10, 2.08 g/day] within the predefined equivalence interval (± 3 g/day). Mean achieved weight remained between 25th -75th reference percentiles of the WHO growth standard through Day 180 by sex and subsequently tracked between 50th -90th percentiles through Day 365. Formula acceptance and tolerance were good. Stool consistency remained soft in both groups throughout the study. No significant group differences in mean fussiness and gassiness scores, or medically confirmed adverse events were detected. A total of 159 participants completed the Day 365 visit (Control, *n* = 82; INV-PHP, *n* = 77).

**Conclusions:**

Overall, partially hydrolyzed cow’s milk protein infant formula with an added prebiotic was safe, well-tolerated, and associated with adequate growth for healthy term infants receiving formula through one year of age.

**Trial registration:**

ClinicalTrials.gov, ClinicalTrials.gov Identifier NCT05047978. Registered 28 August 2021, https://clinicaltrials.gov/study/NCT05047978.

## Background

Breastfeeding is universally recommended as the preferred feeding method for healthy infants [1–3]. When breastfeeding is not possible, infant formulas that have been inspired by the nutritional and functional properties of human milk are available as appropriate substitutes, meet the nutritional requirements of individual infants, and adhere to rigorous regulatory requirements [4, 5]. One type, partially hydrolyzed cow’s milk protein (PHP) formula, has a 30-year global history of safe use [6]. The modified partially hydrolyzed protein [7] has been suggested to be more easily digested and have shorter gastric emptying time compared to intact protein, which may benefit infants with specific digestive sensitivities such as regurgitation, fussiness and gas [8–11]. Whereas PHP formulas are not recommended for allergy management, other gastrointestinal benefits are still being explored [12, 13].

Other compositional modifications in infant formula, such as carbohydrate sources and the addition of functional ingredients, also aim to support infant digestion. For example, reduced lactose content and added dietary fiber have demonstrated potential for managing mild gastrointestinal issues such colic, constipation, and regurgitation [8, 14]. The prebiotic blend of polydextrose (PDX) and galactooligosaccharides (GOS) has been associated with softer stools, a positive impact on the gut microbiota composition, and consolidation of sleep-wake patterns in infants [15–17]. Prebiotics, defined as “substrate that is selectively utilized by host microorganisms conferring a health benefit” [18] are often added to infant formula to simulate the functionality of human milk oligosaccharides.

In a previous growth and tolerance study, an infant formula that had the same PHP source was demonstrated as safe, well-tolerated, and nutritionally suitable [19]. In that study, the formula used as the Control was not an intact cow’s milk protein infant (IP) formula. The present study aimed to assess the growth and tolerance of infants who were randomized to receive an Investigational PHP infant formula or an IP formula (Control) through one year of age. The Investigational formula had lactose at no less than 50% of total carbohydrate and the Control formula had 92% of total carbohydrate as lactose. Both formulas had a prebiotic blend of PDX: GOS (1:1 ratio; 4 g/L). The primary objective was evaluation of rate of weight gain (g/day) from 14 to 120 days of age to establish that the Investigational PHP formula provides suitable growth compared to the Control IP formula. Secondary objectives included evaluation of body weight, length, and head circumference growth rates, achieved anthropometrics, formula intake, tolerance outcomes, quality of life, and medically confirmed adverse events assessed from 14 to approximately 365 days of age.

## Methods

### Study design and participants

The US Food and Drug Administration (FDA) outlines Good Manufacturing Practice (GMP) guidelines to ensure that infant formulas meet the quality standards required to support normal physical growth [5, 20]. To report consistent growth outcomes across different study cohorts, this multicenter, double-blind, randomized, controlled, parallel-group, prospective trial, enrolling healthy 10- to 14-day old infants, recruited at 13 clinical sites in the United States (clinicaltrials.gov: NCT05047978), follows the US FDA GMP guidelines and is similar in design to previously reported studies [21–24]. This study was conducted in compliance with the Declaration of Helsinki, International Council for Harmonisation (ICH) Good Clinical Practice (GCP) guidelines (including October 1996 amendment), and all applicable regulatory requirements. The study protocol and informed consent forms were reviewed and approved by a central Institutional Review Board (IRB), (Advarra, Inc., Columbia, MD). In addition to central approval, each participating site was granted individual site-specific approval by Advarra, Inc. (Columbia, MD). The study complied with good clinical practices. Mothers who had made the decision to exclusively feed infant formula were screened for study eligibility. Parents, guardians, or other legally authorized personnel provided written informed consent prior to enrollment. Inclusion and exclusion criteria were verified (listed in Table 1) and participants were enrolled from August 2021 until study recruitment ended in October 2022. Study visits occurred at 14 (–4 days; randomization), 30 (± 3), 42 (± 3), 60 (± 3), 90 (± 3), 120 (+ 5), 180 (± 7), 275 (± 7), and 365 (± 7) days of age.

**Table 1:** Participant inclusion and exclusion criteria

**Inclusion Criteria**
· 10–14 days of age at randomization, inclusive (day of birth is considered Day 0)
· Singleton birth
· Gestational age of 37–42 weeks (36 weeks and six days is considered 36 weeks gestational age)
· Birth weight of 2500 g (5 lbs 8 oz) or more
· Exclusively receiving infant formula for at least 24 hours prior to randomization
· Parent(s) or legal guardian has full intention to exclusively feed study formula through 120 days of age
· Parent(s) or legal guardian agrees not to enroll infant in another interventional clinical study while participating in this study
· Signed informed consent from parent or legal guardian obtained for infant’s participation in the study
· Signed authorization obtained from parent or legal guardian to use and/or disclose Protected Health Information for infant from birth through the length of the study period
**Exclusion Criteria**
· History of underlying metabolic or chronic disease; congenital malformation; or any other condition which, in the opinion of the Investigator, was likely to interfere with: the ability of the infant to ingest food, the normal growth and development of the infant, or the evaluation of the infant
· Evidence of feeding difficulties or history of formula intolerance, such as vomiting or poor intake, at time of randomization
· Weight at Visit 1 was <98% of birth weight [(weight at Visit 1 ÷ birth weight) x 100 < 98%]
· Infant was born large for gestational age (LGA) (as confirmed by the hospital birth records) from mother who was diabetic at childbirth
· Infant is immunocompromised (according to a doctor’s diagnosis of immunodeficiency such as combined immunodeficiencies, DiGeorge syndrome, Wiskott-Aldrich syndrome, severe congenital neutropenia and secondary immunodeficiencies linked to HIV infection, Down syndrome, or others)

### Randomization and study allocation

Similar to our previous published growth and tolerance studies [21–24], for each study site, the study sponsor created a computer-generated randomization schedule stratified by sex and provided in sealed, opaque, consecutively numbered envelopes. Study site assigned study formula to each participant by opening the next sequential envelope from the appropriate set. Participants were randomly assigned to receive one of two study formulas (Mead Johnson Nutrition, Evansville, IN; Table 2) from Day 14 up to Day 365: intact cow’s milk protein formula (Control; previously marketed Enfamil^®^) or investigational PHP formula (INV-PHP). Both formulas had the prebiotic blend of PDX: GOS. Study formulas were dispensed to parents at each study visit prior to study completion or withdrawal. Each study formula was assigned two unique codes (known only to the study sponsor). Blinding for a participant could be broken by study sponsor personnel in the event of a medical emergency, however, it was not necessary to break the study code prematurely in this study. Parents were instructed to exclusively feed study formula through Day 120;; participants who completed the Day 365 visit were considered to complete the study even if after 180 days of age study formula consumption dropped below two feedings/day or study feeding was discontinued. 

**Table 2:** Nutrient composition per 100 kcal, 20 calories per fl oz^a^

Nutrient	Study Formula (Target Values)	
	Control	INV-PHP
Total Protein, g^b^	1.9	2.3
Total Fat, g	5.5	5.4
Linoleic Acid, mg	800	800
α-Linolenic Acid, mg	67	71
Arachidonic Acid, mg^c^	29	29
Docosahexaenoic Acid, mg^c^	23	23
Total Carbohydrate, g^d^	11	11
Vitamin A, IU	250	250
Vitamin A, mcg	75	75
Vitamin D, IU	70	70
Vitamin E, IU	1.69	2.9
Vitamin K, mcg	5	5
Thiamin, mcg	78	102
Riboflavin, mcg	98	105
Vitamin B6, mcg	35	102
Vitamin B12, mcg	0.15	0.15
Niacin, mcg	550	944
Folate, mcg (Dietary Folate Equivalents; DFE)	18.3	20
Pantothenic Acid, mcg	550	770
Biotin, mcg	3	2
Vitamin C, mg	10	10
Choline, mg	33	33
Inositol, mg	6	6.1
Calcium, mg	78	100
Phosphorus, mg	41	67
Magnesium, mg	8	8
Iron, mg	0.8	0.8
Zinc, mg	0.6	0.7
Manganese, mcg	5.9	10
Copper, mcg	70	71
Iodine, mcg	18	15
Selenium, mcg	3.4	3.5
Sodium, mg	30	39
Potassium, mg	105	131
Chloride, mg	75	75

^a^All nutrients comply with the US Infant Formula Act [5]

^b^Protein sources for Control: intact cow’s milk protein and for INV-PHP: partially hydrolyzed cow’s milk protein (PHP)

^c^Not typically included on label claim panel

^d^Control formula: Includes added prebiotic blend of polydextrose (PDX) and galactooligosaccharides (GOS) (1:1 ratio, 4 g/L), ~92% of carbohydrate as lactose; INV-PHP: Includes added prebiotic blend of PDX and GOS (1:1 ratio, 4 g/L), ~50% of carbohydrate as lactose

### Study outcomes

The primary outcome was rate of weight gain (growth rate; g/day) from 14 to 120 days of age. Anthropometric measures (body weight, length, and head circumference) were recorded at all study visits. Parents completed a 24-hour recall at all study visits that included: fussiness (not fussy = 0/slightly = 1/moderately = 2/very = 3/extremely fussy = 4); amount of gas (none = 0/slight = 1/moderate = 2/excessive = 3); spit-up (number/day); crying (hours/day); stool frequency (number/day); stool consistency (hard = 1/formed = 2/soft = 3/unformed or seedy = 4/watery = 5); difficulty napping (never = 0/almost never = 1/sometimes = 2/often = 3/almost always = 4); night-wakings (number/night); and nighttime sleep quality (very well = 0/well = 1/fairly well = 2/poorly = 3/very poorly = 4); 24-hour recall of study formula intake (fl oz/day) began at Day 30. Adverse events were coded according to specific event and the body system involved.

Parent/caregivers also completed validated, parent-reported questionnaires including the Pediatric Quality of Life Inventory™ Family Impact Module (PedsQL FIM)-Acute at all study visits and the PedsQL Infant Scales-Acute at Days 120, 180, 275, and 365. The 36-item PedsQL FIM-Acute uses a 5-point scale [25] and is reverse-scored and linearly transformed to a 0–100 scale (0 = 100, 4 = 0; higher scores indicate better functioning). Three composite summary scores are reported based on six scales: Parent HRQOL (physical, emotional, social, and cognitive functioning); family functioning (daily activities, family relationships); and total score [25]. The 36-item PedsQL Infant Scales-Acute are also recorded on a 5-point scale, with ranges from 0 (never a problem) to 4 (almost always) for five scales: physical functioning, physical symptoms, emotional functioning, social functioning, and cognitive functioning. Scale scores and total scores were computed in a manner similar to the FIM.

### Statistical analysis

Sample size was chosen to establish equivalence in rate of weight gain from 14 to 120 days (80% power; α = 0.05; two one-tailed) between INV-PHP and Control formulas based on a pre-defined margin of 3 g/day previously specified by American Academy of Pediatrics (AAP) guidelines as a clinically relevant difference [21]. Assuming a standard deviation of 6 g/day and 0.5 g/day difference in weight gain between study formula groups, 78 infants/group were required to complete the Day 120 visit. In allowing for a 35% drop-out rate, approximately 240 participants were enrolled. Sample size was calculated using nQuery + nTerim 3.0 software (Statsols, Boston, MA). Growth rates for pre-specified intervals (14 to 30, 42, 60, 90 and 120 days) were calculated for each participant by fitting a linear regression model of growth measurements on participant age (days). The slope from the regression model was the growth rate.

Secondary outcomes included anthropometrics, tolerance measures, and medically confirmed adverse events through Day 365. Weight, length, and head circumference growth rates were analyzed using Analysis of variance (ANOVA) models with terms for sex, study group and sex-by-study group interaction. In the final model, the sex-by-study group interaction was retained when significant and removed when not significant. When the interaction was significant, post-hoc analysis was performed by ANOVA with a term for study group and each sex analyzed separately. Achieved weight, length, head circumference, and formula intake at Days 30, 42, 60, 90, 120, 180, 275, and 365 were analyzed using an ANOVA model similar to the that used to analyze growth rates. Stool frequency, crying, spit-ups, night-wakings, and PedsQL questionnaire scores were analyzed by ANOVA with a term for study group. Fussiness, gassiness, difficulty napping, nighttime sleep quality, and stool consistency were analyzed by Cochran-Mantel-Haenszel (CMH). Study discontinuation and adverse events were analyzed by Fisher’s exact test.

Analysis of weight gain were based on two one-tailed tests; all other tests were two-tailed (α = 0.05). Statistical analyses were performed using SAS^®^ software (version 9.4; SAS Institute, Cary, NC).

## Results

### Participants

**Fig. 1:**
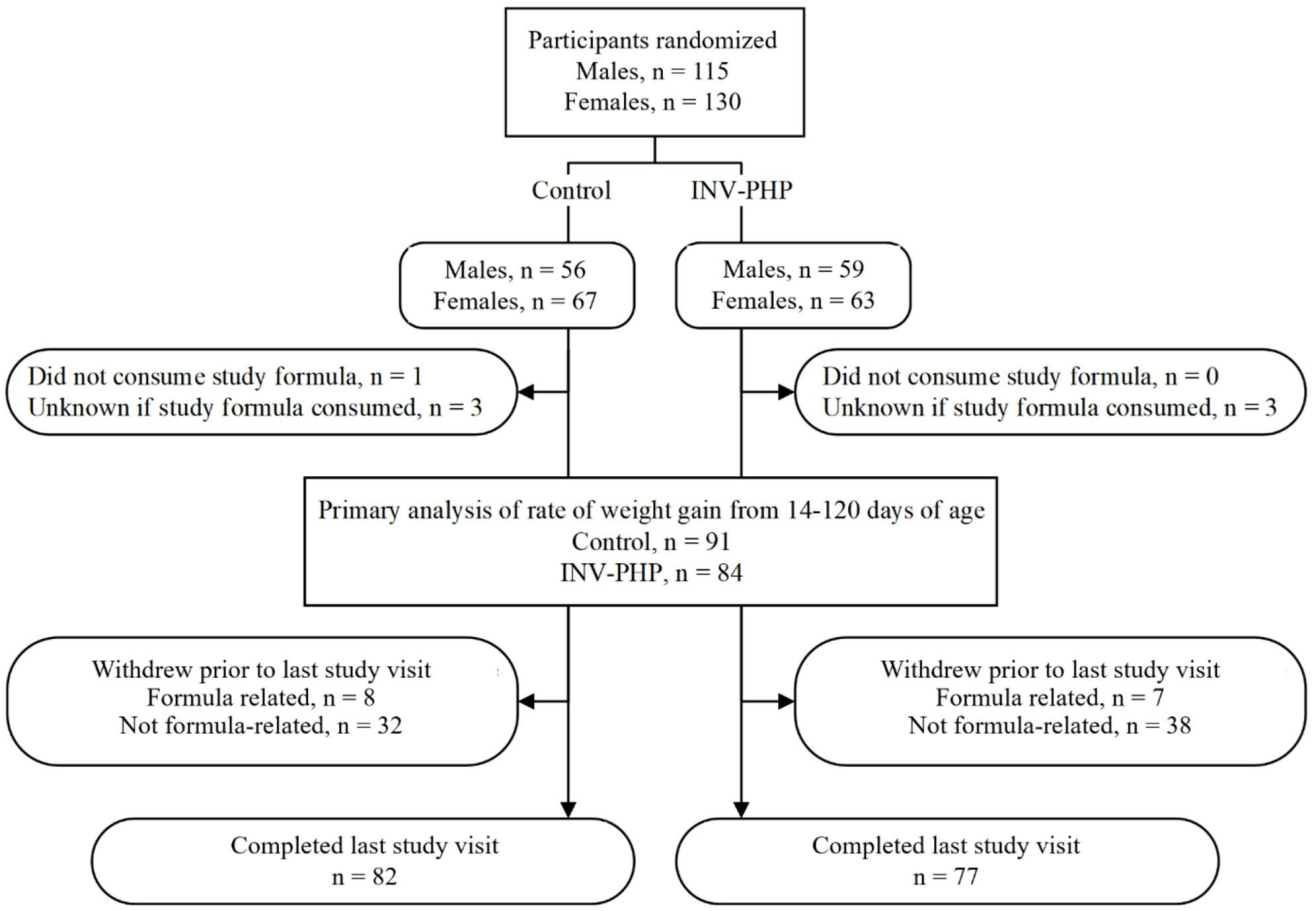
Study allocation flow

A total of 244 infants were enrolled and randomized (Control, *n* = 122; INV-PHP, *n* = 122); 159 completed Day 365 study visit (Control, *n* = 82; INV-PHP, *n* = 77). Participants who were randomized but did not consume study formula (Control, *n* = 1) were excluded from all analyses (Fig. 1). Infant birth anthropometrics and participant, primary caregivers, and family household characteristics were similar between groups at enrollment (Table 3).

**Table 3:** Participant and family characteristics at enrollment

Characteristic	Control	INV-PHP	*P*
Birth characteristics			
Weight (g)^a^	3422.6 ± 35.8	3365.2 ± 47.9	0.257
Sex, n (%)			0.798
Female	66 (54)	63 (52)	
Male	56 (46)	59 (48)	
Race, n (%)			0.646
Black	16 (13)	16 (13)	
White	99 (82)	103 (84)	
More than one race	6 (5)	3 (2)	
Ethnicity, n (%)			0.611
Hispanic	23 (19)	19 (16)	
Not Hispanic	99 (81)	103 (84)	
Feeding history prior to study entry			
Received any human milk since birth, n (%)			0.195
Yes	46 (38)	57 (47)	
No	76 (62)	65 (53)	
Days of exclusive human milk feeding^a^	2.6 ± 0.4	2.4 ± 0.4	0.788
Days of partial human milk feeding^a^	3.7 ± 0.6	4.1 ± 0.5	0.547
Days of exclusive formula feeding^a^	10.7 ± 0.3	10.4 ± 0.3	0.532
Type of formula used prior to study entry, n	99	101	--^b^
Cow’s milk based	2	1	
Soy based	24	21	
Partially hydrolyzed	0	1	
Extensively hydrolyzed			
Gestational Age^a^	38.6 ± 0.1	38.6 ± 0.1	0.543
Mother’s age when infant was born or legally adopted^a^	27.3 ± 0.5	27.5 ± 0.5	0.752
Parity of mother^a^	2.1 ± 0.1	1.9 ± 0.1	0.396
Number of people in household^a^	4.3 ± 0.1	3.9 ± 0.1	0.011
Number of adult caregivers^a^	2.1 ± 0.0	2.0 ± 0.0	0.285
Number of children^a^	2.1 ± 0.1	1.8 ± 0.1	0.045
Mode of delivery, n (%)			0.602
Vaginal	75 (61)	70 (57)	
Cesarean	47 (39)	52 (43)	
First infant for whom you have been the primary caregiver, n (%)			0.297
Yes	45 (37)	54 (44)	
No	77 (63)	68 (56)	
Anyone in the home smokes			0.727
Yes	18 (15)	20 (17)	
No	104 (85)	101 (83)	
Mother’s education level, n (%)			0.785
Partial high school	5 (4)	4 (3)	
High school/GED	38 (31)	40 (33)	
Partial college	21 (17)	27 (22)	
Associate’s degree	15 (12)	11 (9)	
Bachelor’s degree	28 (23)	24 (20)	
Graduate or professional degree	11 (9)	12 (10)	
Unknown	2 (2)	0 (0)	
Prefer not to answer	2 (2)	4 (3)	
Father’s education level, n (%)			0.315
Partial high school	10 (8)	4 (3)	
High school/GED	41 (34)	52 (43)	
Partial college	21 (17)	28 (23)	
Associate’s degree	9 (7)	11 (9)	
Bachelor’s degree	25 (20)	11 (9)	
Graduate or professional degree	6 (5)	8 (7)	
Unknown	5 (4)	4 (3)	
Prefer not to answer	5 (4)	4 (3)	
Annual household income, n (%)			0.459
Less than $25,000	13 (11)	21 (17)	
$25,000 to $34,999	27 (22)	18 (15)	
$35,000 to $49,999	17 (14)	11 (9)	
$50,000 to $74,999	15 (12)	20 (16)	
$75,000 to $99,999	16 (13)	30 (25)	
$100,000 to $149,999	15 (12)	11 (9)	
$150,000 or more	8 (7)	6 (5)	
Prefer not to answer	11 (9)	5 (4)	

^a^ Mean ± standard error (SE)

^b^ not tested

### Growth

A total of 175 participants (Control, *n* = 91; INV-PHP, *n* = 84) were included in the primary analysis to evaluate equivalence in weight growth rate from Day 14–120. The mean difference of 0.49 g/day (INV-PHP vs. Control) and 90% CI [lower, upper: −1.10, 2.08 g/day] were within the predefined equivalence of ± 3 g/day from Day14-120 (Table 4) in addition to other measured intervals (Days 14–42, 14–60, and 14–90) with the exception of Day 14–30 [mean group difference = 1.56 g/day, 90% CI (−1.15, 4.28 g/day)]. No significant group differences were detected for mean length or head circumference growth rates for any measured range (Table 5) with the exception of a small but statistically significant difference in length (Control: 0.12 ± 0.003, INV-PHP 0.13 ± 0.003; *P* = 0.018). Post-hoc analyses are reported and described in data tables when a sex-by-study group interaction was observed.

**Table 4:** Mean weight growth rate (g/day) from day 14 to days 30, 42, 60, 90, and 120

Age Interval	Study Group	*n*	Mean	Mean Difference (INV-PHP vs. Control)	(s.e.)	90% CI	
						Lower	Upper
14–30	Control	108	34.8	1.56^a^	(1.6)	−1.15	4.28
	INV-PHP	108	36.4				
14–42	Control	102	35.5	−0.19	(1.4)	−2.44	2.07
	INV-PHP	93	35.3				
14–60	Control	102	33.7	−0.72^bc^	(1.2)	−2.73	1.30
	INV-PHP	93	33.0				
14–90	Control	97	31.0	−0.03^bd^	(1.1)	−1.84	1.78
	INV-PHP	88	31.0				
14–120	Control	91	28.6	0.49^be^	(1.0)	−1.10	2.08
	INV-PHP	84	29.1				

^a^90% CI outside the predefined equivalence of ± 3 g/day

^b^Interaction term for sex*study group was statistically significant (P<0.05) and included in final statistical model

^c^Post-hoc analysis for Day 14–60 by sex: no significant differences by group

^d^Post-hoc analysis for Day 14–90 by sex: males, no significant different by group/females, Control

^e^Post-hoc analysis for Day 14–120 by sex: males, no significant different by group/females, Control

**Table 5:** Length and head circumference growth rates from 14 days to 30, 60, 90, and 120 days of age

		Growth rate (cm/day)^a^	
Day	Group (n)	Length	Head circumference
30	Control (108)	0.13 ± 0.007	0.09 ± 0.004
	INV-PHP (108)	0.14 ± 0.007	0.09 ± 0.004
42	Control (101)	0.13 ± 0.005	0.08 ± 0.003
	INV-PHP (93)	0.14 ± 0.005	0.08 ± 0.003
60	Control (101)	0.12 ± 0.003^bc^	0.07 ± 0.002
	INV-PHP (93)	0.13 ± 0.003	0.07 ± 0.002
90	Control (96)	0.11 ± 0.002	0.06 ± 0.002
	INV-PHP (88)	0.12 ± 0.002	0.06 ± 0.002
120	Control (90)	0.10 ± 0.002	0.05 ± 0.001
	INV-PHP (84)	0.11 ± 0.002	0.06 ± 0.001

^a^Mean ± standard error (SE)

^b^Statistically significant, *P* < 0.05

^c^Interaction term for sex*study group was statistically significant (p-value < 0.05) and included in final statistical model. Post-hoc analysis for mean length growth rate: females, no group differences; males, Control < INV-PHP, *P* < 0.05

**Fig. 2:**
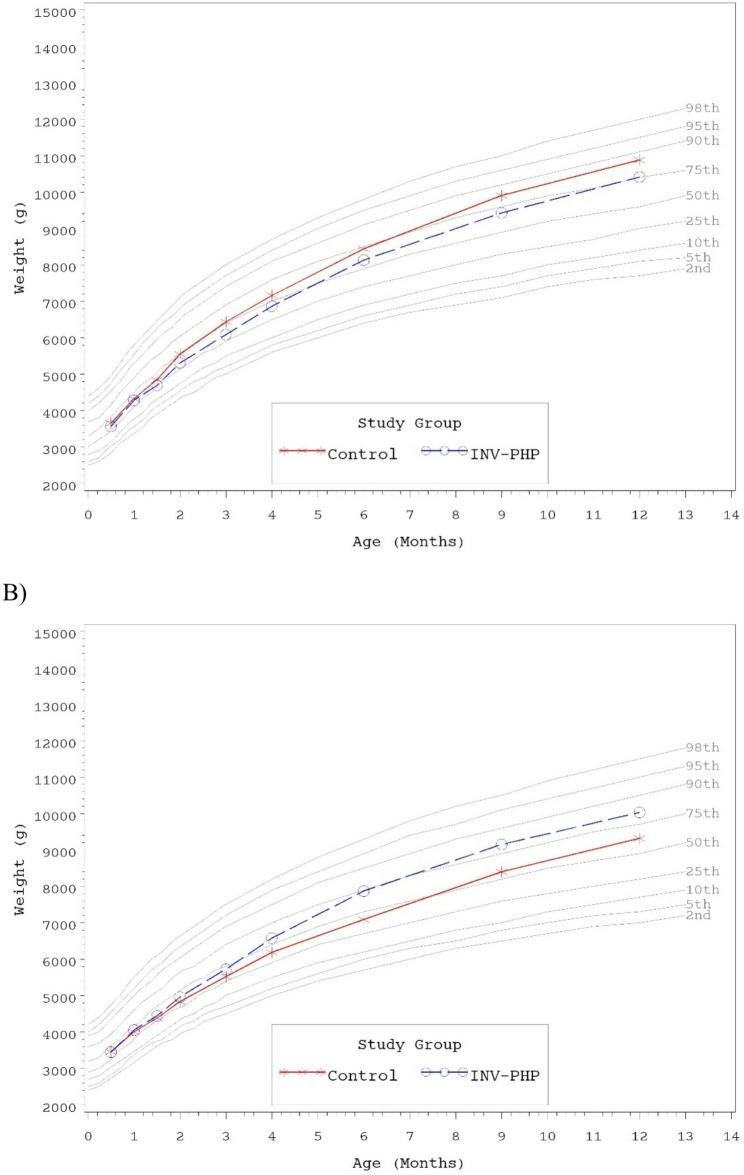
Mean achieved weight for **A**) male participants and **B**) female participants with World Health Organization (WHO) percentiles (2nd to 98th) from 14 to 365 days of age

**Fig. 3:**
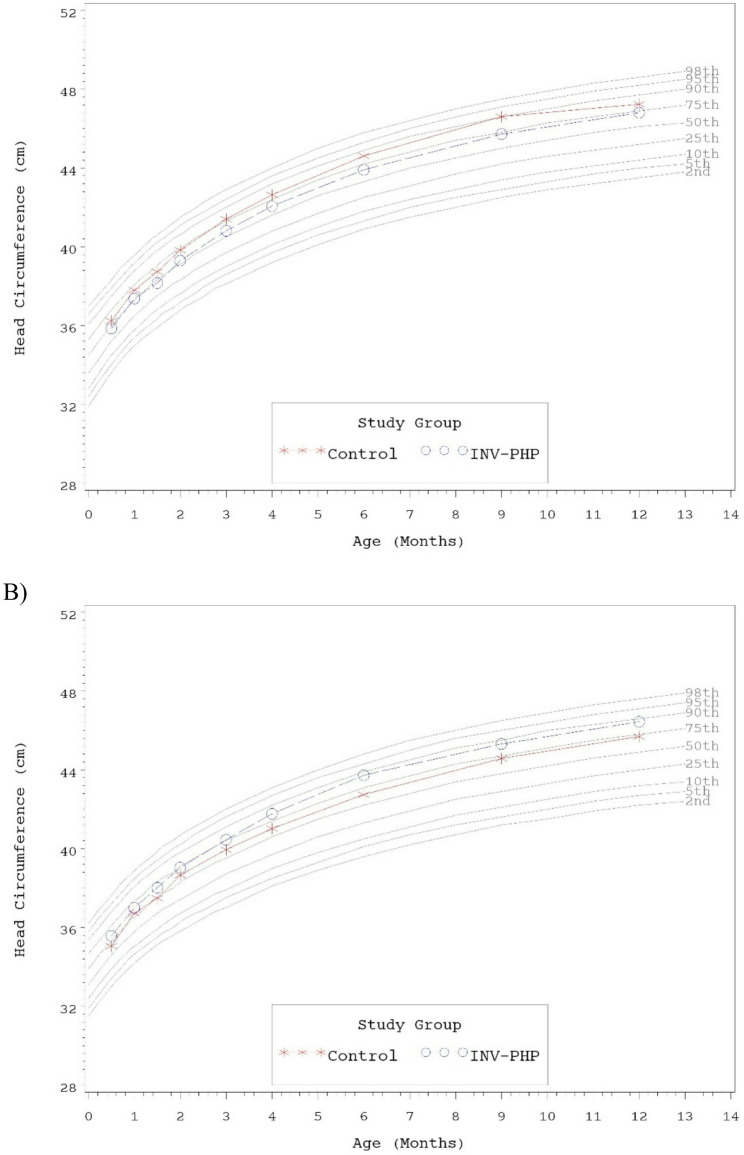
Mean achieved head circumference (HC) for **A**) male participants and **B**) female participants with World Health Organization (WHO) percentiles (2nd to 98th) from 14 to 365 days of age

**Fig. 4:**
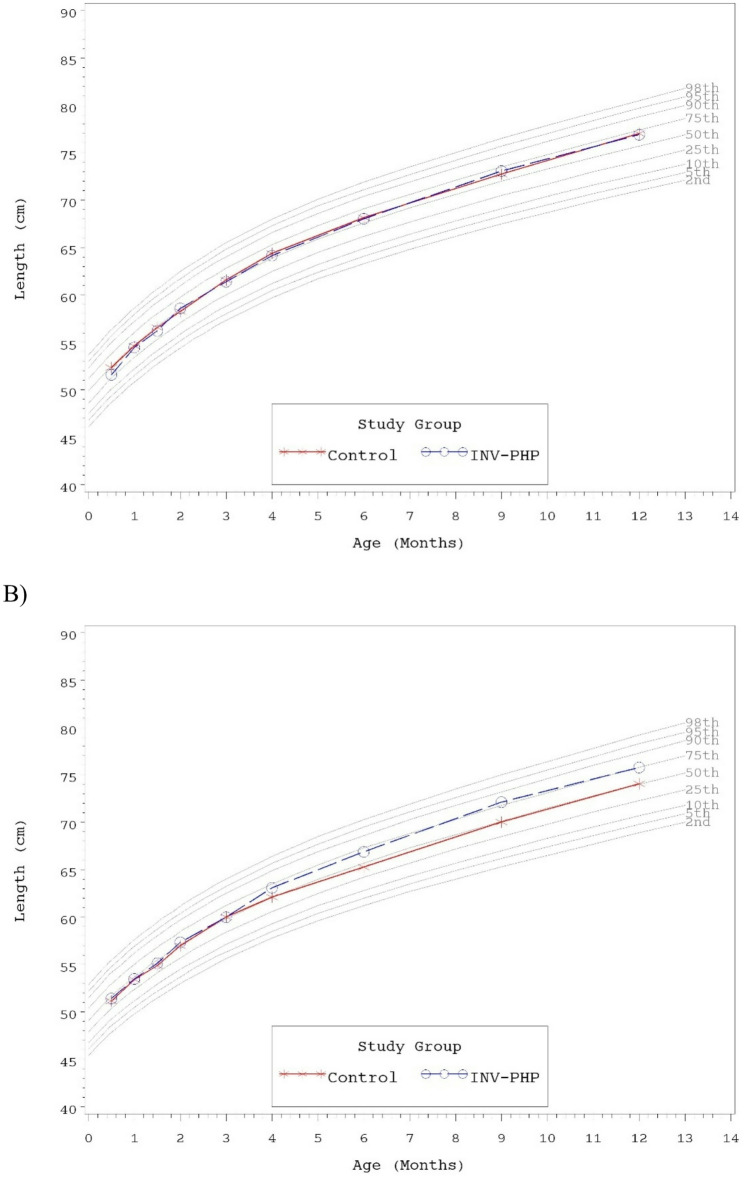
Mean achieved length for **A**) male participants and **B**) female participants with World Health Organization (WHO) percentiles (2nd to 98th) from 14 to 365 days of age

No statistically significant group differences were detected for mean achieved weight, length, or head circumference at any time point assessed, with the exception of significantly lower mean achieved length for Control vs. INV-PHP at Days 180 and 275 (Table 6). Mean achieved weight on the WHO weight-for-age growth standard remained between 25th-75th percentiles through Day 180 for both males and female infants and subsequently tracked between the 50th and 90th percentiles through Day 365 (Fig. 2A and B). Head circumference followed a similar track (Fig. 3A and B). Mean achieved length on the WHO length-for-age growth standard remained between the 25th-75th percentiles through Day 365 for male infants (Fig. 4A). For female infants, achieved length tracked along the 50th percentile through Day 90; subsequently the Control group continued along the 50th percentile and the INV-PHP tracked along the 75th percentile through Day 365 (Fig. 4B). In addition, group differences were not significant for mean weight-for-age or head circumference-for-age z-scores, and only isolated significant group differences were detected in length-for-age or weight-for-length z-scores (Table 7). Mean z-scores appeared to fall within the range described as “normal” (−1 to 1 range) by the WHO guidelines for interpretation of growth indicators [26].

**Table 6:** Achieved weight, length, head circumference (HC), and weight-for-length z-scores at days 30, 60, 90, and 120, for males and females

Age (Day)		Achieved Growth^a^		
	Group (*n*)	Weight (g)^d^	Length (cm)^e^	HC (cm)^f^
30	Control (108)	4170 ± 42	54.0 ± 0.2	37.3 ± 0.1^c^
	INV-PHP (108)	4170 ± 42	54.0 ± 0.2	37.2 ± 0.1
42	Control (101)	4613 ± 48	55.7 ± 0.2	38.1 ± 0.1^c^
	INV-PHP (93)	4576 ± 50	55.7 ± 0.2	38.1 ± 0.1
60	Control (101)	5197 ± 56^c^	57.6 ± 0.2	39.2 ± 0.1^c^
	INV-PHP (91)	5137 ± 59	58.0 ± 0.2	39.2 ± 0.1
90	Control (95)	5980 ± 68^c^	60.8 ± 0.3	40.7 ± 0.1^c^
	INV-PHP (84)	5907 ± 72	60.7 ± 0.2	40.6 ± 0.1
120	Control (87)	6674 ± 82^c^	63.2 ± 0.2	41.8 ± 0.1^c^
	INV-PHP (80)	6722 ± 85	63.6 ± 0.3	41.9 ± 0.1
180	Control (88)	7768 ± 97^c^	66.7 ± 0.2^bc^	43.7 ± 0.1^c^
	INV-PHP (81)	8002 ± 101	67.5 ± 0.2	43.8 ± 0.1
275	Control (85)	9154 ± 110^c^	71.4 ± 0.2^bc^	45.6 ± 0.1^c^
	INV-PHP (77)	9290 ± 116	72.6 ± 0.3	45.5 ± 0.1
365	Control (81)	10,101 ± 122^c^	75.5 ± 0.3^c^	46.7 ± 0.1^c^
	INV-PHP (78)	10,221 ± 124	76.3 ± 0.3	46.6 ± 0.1

^a^Mean ± standard error (SE)

^b^Statistically significant, *P* < 0.05

^c^Interaction term for sex*study group was statistically significant (p-value <0.05) and included in final statistical model

^d^Post-hoc analysis for mean achieved weight by sex: Day 60: no group differences; Day 90: females, no group differences/males, Control > INV-PHP, P=0.05; Days 120, 180, 275, and 365: males, no group differences/females Control

^e^Post-hoc analysis for mean achieved length by sex: Days 180, 275, and 365: males, no group differences/females, Control< INV-PHP, all P<0.05

^f^Post-hoc analysis for mean achieved HC by sex: Day 30: no group differences; Days 42, 60, 90, 120, 275, 365: males, Control>INV-PHP, P<0.05; Days 42, 90, 120, 180, 275, and 365: females, Control< INV-PHP, P<0.05

**Table 7:** Weight-for-age, Length-for-age, and Weight-for-length z-scores at days 0, 14, 30, 42, 60, 90, 120, 180, 275, and 365^a^

Age (Day)	Study Group	*n*	Weight-for-age^e^		Length-for-age^f^		Weight-for-length^g^		Head circumference-for-age^h^		
0	Control	122	0.3	(0.1)	0.8	(0.1)	−0.5	(0.1)	0.3	(0.1)	
	INV-PHP	122	0.1	(0.1)	0.7	(0.1)	−0.7	(0.1)	0.2	(0.1)	
14	Control	121^b^	−0.1	(0.1)	0.0	(0.1)	−0.6	(0.1)	0.2	(0.1)	
	INV-PHP	123	−0.2	(0.1)	−0.1	(0.1)	−0.6	(0.1)	0.2	(0.1)	
30	Control	108	−0.3	(0.1)	−0.1	(0.1)	−0.4	(0.1)	0.3	(0.1)	
	INV-PHP	108	−0.3	(0.1)	−0.1	(0.1)	−0.3	(0.1)	0.3	(0.1)	
42	Control	101	−0.3	(0.1)	0.0	(0.1)	−0.4	(0.1)	0.4	(0.1)	
	INV-PHP	93	−0.3	(0.1)	0.0	(0.1)	−0.4	(0.1)	0.3	(0.1)	
60	Control	101	−0.3^d^	(0.1)	−0.1	(0.1)	−0.2^cd^	(0.1)	0.5	(0.1)	
	INV-PHP	91	−0.4	(0.1)	0.1	(0.1)	−0.6	(0.1)	0.4	(0.1)	
90	Control	95	−0.2^d^	(0.1)	0.1	(0.1)	−0.3^d^	(0.1)	0.6	(0.1)	
	INV-PHP	84	−0.3	(0.1)	0.1	(0.1)	−0.4	(0.1)	0.6	(0.1)	
120	Control	87	−0.1^d^	(0.1)	0.1	(0.1)	−0.2^d^	(0.1)	0.6	(0.1)	
	INV-PHP	80	−0.0	(0.1)	0.3	(0.1)	−0.3	(0.1)	0.7	(0.1)	
180	Control	88	0.1^d^	(0.1)	0.1^cd^	(0.1)	0.2^d^	(0.1)	0.8	(0.1)	
	INV-PHP	81	0.4	(0.1)	0.4	(0.1)	0.3	(0.1)	0.9	(0.1)	
275	Control	85	0.5^d^	(0.1)	0.1^cd^	(0.1)	0.7^d^	(0.1)	0.9	(0.1)	
	INV-PHP	77	0.6	(0.1)	0.6	(0.1)	0.5	(0.1)	0.8	(0.1)	
365	Control	81	0.7^d^	(0.1)	0.2	(0.1)	0.7^d^	(0.1)	0.9	(0.1)	
	INV-PHP	78	0.7	(0.1)	0.5	(0.1)	0.7	(0.1)	0.8	(0.1)	

^a^Mean ± standard error (SE)

^b^*n* = 120 for Length-for-age and Weight-for-length

^c^Statistically significant, *P* < 0.05

^d^Interaction term for sex*study group was statistically significant (p-value <0.05) and included in final statistical model

^e^Post-hoc analysis for Weight-for-age by sex: males, Days 60, 90 275, 365, Control > INV-PHP, P<0.05/females, Days 180, 365, Control < INV-PHP, P<0.05

^f^Post-hoc analysis for Length-for-age by sex:males, no significant group differences at Days 180, 275/females, Control

^g^Post-hoc analysis for Weight-for-length by sex: males, Days 60, 90, 275, 365, Control>INV-PHP, P<0.05/females, Days 180, 365, Control

^h^Post-hoc analysis for Head circumference-for-age by sex: males, Days 42, 60, 90, 120, 180, 275. 365, Control > INV-PHP, P<0.05/females, Days 14, 42, 90, 120, 180, 275, 365, Control < INV-PHP, P<0.05

### Tolerance

Mean duration (days) of study feeding was similar between groups (Control, 271 ± 12.9; INV-PHP, 253 ± 12.9; *P* = 0.320). Mean study formula intake at Day 30, 42, 60, 90, 120, 180, and 275 (Table 8) were comparable between study groups. Within each study group, mean study formula intake generally increased Day 30 through day 180 and subsequently leveled off or decreased with participant age. By Day 365, study formula intake was lower in both groups and significantly lower in the Control vs. INV-PHP group (*P* = 0.025). There was no group difference for intake of complementary feeding were observed (Table 9). However, amounts of complementary feeding were low (typically in the “teaspoon” range) through Day 90 and then appeared to increase in both groups at Day 120, likely as participants neared the typical age of introduction of complementary foods (between approximately 4 to 6 months of age). Group differences in parent-reported gassiness and fussiness were not significant at any time point assessed (Table 10). Mean stool frequency was significantly lower in the Control vs. INV-PHP group at Days 30, 60, and 180 (Table 11). A significant group difference in stool consistency was detected at Day 30 (*P* = 0.042); in the Control vs. INV-PHP group, fewer infants with “unformed or seedy” and “watery” and more infants with “formed” or “soft” stool consistency were the primary differences observed in stool consistency categories. At all other measured timepoints, stool consistency across categories was similar between groups (Table 11). No significant group differences in crying or spit-up were detected during the study, with the exception of significantly less crying at Day 30 in INV-PHP vs. Control group (Table 12).

**Table 8:** Mean study formula intake (fl oz/day) at days 30, 42, 60, 90, 120, 180, 275, and 365

Age (Day)	Study Group	*n*	Mean	(s.e.)	*P*
30	Control	105	26.8	(0.8)	0.835
	INV-PHP	103	27.0	(0.8)	
42	Control	100	30.5	(0.8)	0.214
	INV-PHP	92	29.1	(0.8)	
60	Control	101	32.1	(0.9)	0.185
	INV-PHP	89	30.5	(0.9)	
90	Control	95	34.6	(1.2)	0.410
	INV-PHP	84	33.1	(1.3)	
120	Control	87	34.8	(1.2)	0.953
	INV-PHP	80	35.0	(1.3)	
180	Control	88	37.0	(1.4)	0.697
	INV-PHP	81	36.2	(1.5)	
275	Control	84	36.5	(1.4)	0.230
	INV-PHP	78	34.1	(1.4)	
365	Control	76	24.3	(1.5)	0.025*
	INV-PHP	72	29.1	(1.5)	

*Statistically significant, p-value < 0.05

**Table 9:** Intake of complementary foods since the previous study visit

Age (Days)	Intake of Complementary Food	Study Group				*p*-value
		Control		INV-PHP		
		n	(%)	n	(%)	
30	Yes	1	(1)	2	(2)	0.620
	No	105	(99)	102	(98)	
42	Yes	2	(2)	3	(3)	0.673
	No	98	(98)	90	(97)	
60	Yes	2	(2)	3	(3)	0.664
	No	98	(98)	83	(97)	
90	Yes	7	(7)	4	(5)	0.545
	No	88	(93)	80	(95)	
120	Yes	14	(16)	11	(14)	0.828
	No	72	(84)	67	(86)	

**Table 10:** Fussiness and gassiness at days 14, 30, 60, 90, 120, 180, 275, and 365^a, b^

Age (Day)	Group	Fussiness, *n* (%)					*P*	Gassiness, *n* (%)				*P*
		Not at all	Slightly	Moderately	Very	Extremely		None at all	Slight amount	Moderate amount	Excessive amount	
14	Control	24 (20)	73 (60)	20 (16)	4 (3)	1 (1)	0.557	14 (11)	45 (37)	59 (48)	4 (3)	0.313
	INV-PHP	25 (20)	63 (52)	29 (24)	5 (4)	0 (0)		12 (10)	43 (35)	57 (47)	10 (8)	
30	Control	13 (12)	68 (65)	19 (18)	5 (5)	0 (0)	0.948	3 (3)	32 (30)	57 (54)	13 (12)	0.818
	INV-PHP	18 (17)	59 (57)	20 (19)	5 (5)	1 (1)		7 (7)	29 (28)	51 (50)	16 (16)	
42	Control	17 (17)	51 (51)	27 (27)	3 (3)	2 (2)	0.718	4 (4)	27 (27)	61 (61)	8 (8)	0.584
	INV-PHP	12 (13)	52 (57)	20 (22)	5 (5)	2 (2)		5 (5)	31 (34)	45 (49)	11 (12)	
60	Control	17 (17)	58 (57)	22 (22)	3 (3)	1 (1)	0.454	6 (6)	30 (30)	53 (52)	12 (12)	0.272
	INV-PHP	20 (22)	48 (53)	20 (22)	1 (1)	1 (1)		3 (3)	37 (41)	44 (49)	6 (7)	
90	Control	29 (31)	51 (54)	12 (13)	3 (3)	0 (0)	0.426	9 (9)	40 (42)	41 (43)	5 (5)	0.481
	INV-PHP	22 (26)	47 (56)	11 (13)	3 (4)	1 (1)		4 (5)	48 (57)	29 (35)	3 (4)	
120	Control	21 (24)	46 (53)	14 (16)	5 (6)	1 (1)	0.191	10 (11)	36 (41)	36 (41)	5 (6)	0.644
	INV-PHP	26 (33)	39 (49)	10 (13)	4 (5)	0 (0)		9 (12)	36 (46)	29 (37)	4 (5)	
180	Control	29 (33)	39 (44)	17 (19)	3 (3)	0 (0)	0.705	22 (25)	35 (40)	29 (33)	2 (2)	0.416
	INV-PHP	17 (21)	51 (63)	11 (14)	2 (2)	0 (0)		13 (16)	40 (50)	23 (29)	4 (5)	
275	Control	32 (38)	34 (49)	13 (15)	3 (4)	2 (2)	0.459	26 (31)	45 (53)	13 (15)	1 (1)	0.648
	INV-PHP	15 (19)	49 (63)	12 (15)	2 (3)	0 (0)		26 (33)	32 (41)	20 (26)	0 (0)	
365	Control	31 (41)	34 (45)	9 (12)	0 (0)	2 (3)	0.942	29 (39)	33 (44)	13 (17)	0 (0)	0.469
	INV-PHP	22 (30)	45 (62)	6 (8)	0 (0)	0 (0)		35 (48)	25 (34)	13 (18)	0 (0)	

^a^24-hour recall at study visits

^b^Mean ± standard error (SE)

**Table 11:** Stool characteristics at days 14, 30, 60, 90, 120, 180, 275, and 365^a^

Age (Days)	Group (*n*)	Stool frequency^b^	*P*	Stool consistency, *n* (%)						*P*
				hard	formed	soft		unformed or seedy	watery	
14	Control (122)	3.2 ± 0.2	0.677	0 (0)	4 (3)	58 (49)	54 (45)		3 (3)	0.791
	INV-PHP (121)	3.1 ± 0.2		0 (0)	3 (3)	56 (48)	54 (47)		3 (3)	
30	Control (104)	2.5 ± 0.2	0.016*	1 (1)	7 (7)	60 (58)	34 (33)		1 (1)	0.042*
	INV-PHP (103)	3.0 ± 0.2		3 (3)	1 (1)	52 (50)	39 (38)		8 (8)	
42	Control (99)	2.4 ± 0.2	0.191	0 (0)	4 (4)	61 (62)	29 (30)		4 (4)	0.326
	INV-PHP (91)	2.7 ± 0.2		0 (0)	1 (1)	55 (60)	32 (35)		4 (4)	
60	Control (100)	2.1 ± 0.2	0.019*	0 (0)	1 (1)	59 (60)	36 (36)		3 (3)	0.854
	INV-PHP (89)	2.6 ± 0.2		0 (0)	3 (3)	52 (59)	28 (32)		5 (6)	
90	Control (94)	2.2 ± 0.2	0.334	0 (0)	2 (2)	56 (64)	28 (32)		2 (2)	0.969
	INV-PHP (82)	2.5 ± 0.2		0 (0)	2 (2)	56 (67)	20 (24)		5 (6)	
120	Control (84)	2.0 ± 0.1	0.131	1 (1)	2 (2)	59 (72)	17 (21)		3 (4)	0.744
	INV-PHP (78)	2.3 ± 0.1		0 (0)	4 (5)	61 (76)	10 (13)		5 (6)	
180	Control (85)	2.0 ± 0.1	0.028*	0 (0)	11 (13)	56 (68)	14 (17)		1 (1)	0.525
	INV-PHP (79)	2.4 ± 0.1		0 (0)	12 (15)	61 (75)	4 (5)		4 (5)	
275	Control (84)	2.1 ± 0.1	0.255	4 (5)	20 (24)	54 (64)	4 (5)		2 (2)	0.455
	INV-PHP (76)	2.3 ± 0.1		4 (5)	24 (31)	44 (57)	3 (4)		2 (3)	
365	Control (73)	2.0 ± 0.1	0.128	3 (4)	36 (48)	33 (44)	11 (1)		2 (3)	0.615
	INV-PHP (69)	2.2 ± 0.1		2 (3)	30 (41)	39 (53)	2 (3)		0 (0)	

^a^24-hour recall at study visits

^b^Mean ± standard error (SE)

*Statistically significant, *P* < 0.05

**Table 12:** Spit-up and crying at days 14, 30, 42, 60, 90, 120, 180, 275, and 365^a^

Age (Days)	Study Group	Spit-up (number/day)			*P*	Crying (hours/day)			*P*
		*n*	Mean	(s.e.)		*n*	Mean	(s.e.)	
14	Control	122	2.3	(0.2)	0.633	122	2.4	(0.2)	0.769
	INV-PHP	122	2.5	(0.2)		121	2.5	(0.2)	
30	Control	103	3.6	(0.4)	0.044*	105	3.7	(0.3)	0.089
	INV-PHP	97	2.6	(0.4)		100	2.8	(0.4)	
42	Control	96	3.8	(0.4)	0.362	99	4.0	(0.4)	0.325
	INV-PHP	87	3.3	(0.4)		91	3.5	(0.4)	
60	Control	101	4.0	(0.4)	0.156	101	4.1	(0.4)	0.289
	INV-PHP	85	3.2	(0.4)		90	3.5	(0.4)	
90	Control	92	3.8	(0.4)	0.237	94	3.8	(0.4)	0.263
	INV-PHP	84	3.1	(0.4)		84	3.2	(0.4)	
120	Control	85	3.9	(0.4)	0.271	85	4.0	(0.4)	0.230
	INV-PHP	78	3.3	(0.4)		80	3.3	(0.4)	
180	Control	88	2.6	(0.3)	0.286	88	2.7	(0.3)	0.370
	INV-PHP	79	2.2	(0.3)		81	2.4	(0.3)	
275	Control	85	0.8	(0.1)	0.453	85	0.8	(0.1)	0.305
	INV-PHP	76	1.0	(0.1)		78	1.0	(0.1)	
365	Control	76	0.3	(0.2)	0.520	76	0.3	(0.2)	0.401
	INV-PHP	71	0.5	(0.2)		72	0.5	(0.2)	

^a^24-hour recall at study visits

*Statistically significant, *P* < 0.05

There were no statistically significant group differences found for overall discontinuation of study either related (Control: 8, 7%; INV-PHP: 7, 6%) or not related to study formula (Control: 32, 26%; INV-PHP: 38, 31%). For formula-related discontinuation, intolerance to study formula determined by the study investigator was the most common reason (Control: 7; INV-PHP: 7) with vomiting (Control: 4; INV-PHP: 2) and fussiness (Control: 2; INV-PHP: 3) as the most commonly reported feeding intolerance. Parental decision was the most common reason for discontinuation not related to study formula (Control: 12; INV-PHP: 21). The number of participants who had at least one medically confirmed adverse event reported was not significantly different between groups (Control: 105/122, 86%; INV-PHP: 102/122, 84%; *P* = 0.722). No group differences were detected in the overall incidence of adverse events by body system including: Body as a Whole; Cardiovascular; Eye, Ears, Nose, and Throat; Gastrointestinal (GI); Metabolic and Nutrition; Musculoskeletal; Respiratory; Skin; and Urogenital, the incidence of any specific adverse events within any body system, or in the number of participants who had allergic conditions or a GI infection. Any medically confirmed adverse event was considered serious if it met one or more of the following criteria: resulted in death, was life-threatening, required inpatient hospitalization or prolongation of existing hospitalization, resulted in persistent or significant disability/incapacity, or was a congenital anomaly/birth defect. A total of 26 participants experienced serious adverse events (Control: 12/122, 10%; INV-PHP: 14/122, 11%). No serious adverse events were considered to be related to study formula; one was undetermined, and all others were deemed unrelated to study formula.

### Quality of life

There were no significant group differences in sleep characteristics: difficulty napping, night-wakings, and nighttime sleep quality during the study with one exception at Day 90 for nighttime sleep quality [primary differences for Control vs. INV-PHP were more infants reported with “well” (20 vs. 12%) and fewer infants with “fairly well” (5 vs. 20%)] (Table 13). For Quality of Life questionnaires, PedsQL FIM scores were comparable between groups (Table 14), with the exception of a significantly lower mean score for Family Functioning Summary Score at baseline (Day 14) and Family Relationships at Day 30 for Control vs. INV-PHP. No significant differences were detected between study formula groups in PedsQL Infant Scales-Acute scores (Table 15), with the exception of a significantly higher mean score at day 275 for Physical Symptoms Score and Physical Health Summary Score for the Control compared to INV-PHP group.

**Table 13:** Night-wakings, nighttime sleep quality, and difficulty napping at days 14, 30, 42, 60, 90, 120, 180 275, and 365^a^

Age (Days)	Group	*n*	Night-wakings^b^	*P*	Nighttime Sleep Quality, *n* (%)					*P*	Difficulty Napping, *n* (%)					*P*
					Very Well	Well	Fairly Well	Poorly	Very Poorly		Never	Almost never	Sometimes	Often	Almost always	
14	Control	120	2.7 ± 0.1	0.521	46 (38)	40 (33)	27 (22)	9 (7)	0 (0)	0.947	67 (55)	32 (26)	17 (14)	4 (3)	2 (2)	0.616
	INV-PHP	122	2.8 ± 0.1		48 (39)	35 (29)	30 (25)	9 (7)	0 (0)		57 (47)	41 (34)	20 (16)	4 (3)	0 (0)	
30	Control	104	2.3 ± 0.1	0.359	42 (40)	40 (38)	22 (21)	1 (1)	0 (0)	0.849	40 (38)	34 (32)	28 (27)	3 (3)	0 (0)	0.887
	INV-PHP	102	2.4 ± 0.1		48 (47)	35 (34)	12 (12)	8 (8)	0 (0)		42 (41)	30 (29)	26 (25)	3 (3)	2 (2)	
42	Control	100	1.9 ± 0.1	0.864	42 (42)	35 (35)	22 (22)	1 (1)	0 (0)	0.560	36 (36)	36 (36)	26 (26)	1 (1)	1 (1)	0.385
	INV-PHP	92	2.0 ± 0.1		44 (48)	31 (34)	13 (14)	4 (4)	0 (0)		30 (33)	35 (38)	19 (21)	7 (8)	1 (1)	
60	Control	99	1.6 ± 0.1	0.112	58 (58)	27 (27)	11 (11)	4 (4)	0 (0)	0.992	37 (37)	36 (36)	19 (19)	9 (9)	0 (0)	1.000
	INV-PHP	88	1.3 ± 0.1		51 (57)	23 (26)	16 (18)	0 (0)	0 (0)		35 (39)	25 (28)	26 (29)	3 (3)	1 (1)	
90	Control	95	1.1 ± 0.1	0.730	70 (74)	19 (20)	5 (5)	1 (1)	0 (0)	0.047*	44 (46)	32 (34)	14 (15)	4 (4)	1 (1)	0.141
	INV-PHP	83	1.0 ± 0.1		56 (67)	10 (12)	17 (20)	1 (1)	0 (0)		29 (35)	28 (34)	23 (28)	3 (4)	0 (0)	
120	Control	85	1.1 ± 0.1	0.122	55 (63)	14 (16)	14 (16)	4 (5)	0 (0)	0.496	38 (44)	22 (25)	18 (21)	7 (8)	2 (2)	0.128
	INV-PHP	79	0.9 ± 0.1		55 (69)	12 (15)	10 (13)	2 (3)	1 (1)		39 (49)	26 (33)	10 (13)	5 (6)	0 (0)	
180	Control	88	1.2 ± 0.1	0.111	48 (55)	21 (24)	14 (16)	5 (6)	0 (0)	0.358	33 (38)	23 (26)	25 (28)	6 (7)	1 (1)	0.204
	INV-PHP	81	0.9 ± 0.1		46 (57)	22 (27)	12 (15)	1 (1)	0 (0)		35 (43)	24 (30)	18 (22)	4 (5)	0 (0)	
275	Control	82	1.1 ± 0.1	0.210	44 (52)	24 (28)	14 (16)	2 (2)	1 (1)	0.402	46 (55)	16 (19)	18 (21)	3 (4)	1 (1)	0.504
	INV-PHP	77	0.8 ± 0.1		44 (56)	23 (29)	8 (10)	3 (4)	0 (0)		32 (41)	27 (35)	16 (21)	3 (4)	0 (0)	
365	Control	76	0.8 ± 0.1	0.105	47 (62)	13 (17)	12 (16)	4 (5)	0 (0)	0.338	39 (51)	18 (24)	18 (24)	0 (0)	1 (1)	0.881
	INV-PHP	73	0.6 ± 0.1		49 (67)	13 (18)	9 (12)	2 (3)	0 (0)		40 (55)	19 (26)	9 (12)	3 (4)	2 (3)	

^a^24-hour recall at study visits

^b^Mean ± standard error (SE)

*Statistically significant, *P* < 0.05

**Table 14:** PedsQL Family Impact Module (FIM) - Acute scores at days 14, 30, 42, 60, 90, 120, 180, 275, and 365

Age (Days)	Study Group	*n*	Physical Functioning	Emotional Functioning	Social Functioning	Cognitive Functioning	Communication	Worry	Daily Activities	Family relationships	Parent HRQOL Summary Score	Family Functioning Summary Score^g^	Total Scale Score
14	Control	122	71.7 ± 1.3	87.5 ± 1.4	88.7 ± 1.4	82.2 ± 1.8	94.9 ± 1.0	91.6 ± 1.1	73.2 ± 2.0	91.6 ± 1.2	81.7 ± 1.2	84.7 ± 1.3^*^	84.8 ± 1.0
	INV-PHP	122	72.2 ± 1.3	86.8 ± 1.4	90.0 ± 1.4	85.4 ± 1.8	96.1 ± 1.0	91.3 ± 1.1	78.4 ± 2.0	94.6 ± 1.2	82.7 ± 1.2	88.5 ± 1.3	86.3 ± 1.0
30	Control	105	75.4 ± 1.5	86.5 ± 1.4	89.4 ± 1.5	83.6 ± 1.7	95.6 ± 0.9	93.2 ± 1.2^b^	73.2 ± 2.4^b^	92.8 ± 1.1^b*^	83.0 ± 1.3	86.3 ± 1.2^b^	86.0 ± 1.1
	INV-PHP	103	78.7 ± 1.5	90.0 ± 1.4	91.3 ± 1.5	87.1 ± 1.8	97.4 ± 0.9	91.7 ± 1.2	76.6 ± 2.4	96.1 ± 1.1	86.1 ± 1.3	88.0 ± 1.2	88.4 ± 1.1
42	Control	100	80.6 ± 1.5	89.5 ± 1.5	91.9 ± 1.4	84.6 ± 1.9	96.3 ± 0.9^a^	92.9 ± 1.3	79.6 ± 2.4	94.1 ± 1.2	86.1 ± 1.4	89.1 ± 1.2	88.4 ± 1.1
	INV-PHP	92	81.3 ± 1.6	89.6 ± 1.6	91.4 ± 1.4	87.2 ± 2.0	97.2 ± 0.9	91.4 ± 1.4	77.8 ± 2.5	94.6 ± 1.2	86.8 ± 1.4	87.8 ± 1.3	88.7 ± 1.2
60	Control	101	80.0 ± 1.6	90.4 ± 1.4	92.6 ± 1.3	85.5 ± 1.8	97.6 ± 0.7	95.0 ± 1.2	79.3 ± 2.2	94.0 ± 1.1	86.5 ± 1.3	88.9 ± 1.1	89.1 ± 1.1
	INV-PHP	90	84.0 ± 1.7	90.9 ± 1.5	91.7 ± 1.4	90.2 ± 1.9	98.1 ± 0.7	92.4 ± 1.3	80.1 ± 2.4	95.6 ± 1.1	88.8 ± 1.4	89.3 ± 1.2	90.3 ± 1.1
90	Control	94	82.2 ± 1.8	89.8 ± 1.6	92.0 ± 1.7	85.2 ± 1.9	96.5 ± 0.8	93.7 ± 1.2^c^	78.9 ± 2.5^c^	93.1 ± 1.3^c^	86.8 ± 1.5	88.4 ± 1.3^c^	88.8 ± 1.3
	INV-PHP	84	82.6 ± 1.9	88.8 ± 1.7	89.3 ± 1.8	88.1 ± 2.0	97.5 ± 0.9	94.9 ± 1.3	79.0 ± 2.6	94.3 ± 1.4	86.9 ± 1.6	87.8 ± 1.3	89.2 ± 1.4
120	Control	86	82.9 ± 1.7	91.1 ± 1.6	91.9 ± 1.6	87.3 ± 2.0	97.1 ± 0.9	96.8 ± 1.2	80.7 ± 2.5^e^	94.3 ± 1.4^c^	87.4 ± 1.6^c^	89.7 ± 1.4^e^	89.9 ± 1.3^e^
	INV-PHP	80	84.4 ± 1.8	88.1 ± 1.7	90.2 ± 1.7	86.4 ± 2.1	97.2 ± 0.9	93.8 ± 1.2	80.5 ± 2.6	93.3 ± 1.5	87.0 ± 1.7	87.9 ± 1.4	89.1 ± 1.4
180	Control	88	84.0 ± 1.7	91.9 ± 1.5	93.0 ± 1.4	87.6 ± 2.0	96.6 ± 0.9	96.5 ± 1.2	81.8 ± 2.4	93.7 ± 1.5^e^	88.7 ± 1.5	89.9 ± 1.5^e^	90.5 ± 1.3
	INV-PHP	81	84.6 ± 1.8	89.9 ± 1.6	93.8 ± 1.5	88.8 ± 2.1	98.1 ± 0.9	94.3 ± 1.3	82.0 ± 2.5	95.8 ± 1.6	88.8 ± 1.6	89.9 ± 1.5	90.8 ± 1.3
275	Control	85	84.0 ± 1.7	91.4 ± 1.6	93.8 ± 1.5	88.2 ± 1.9	97.6 ± 0.9	98.1 ± 0.8	81.5 ± 2.3	93.9 ± 1.5	88.8 ± 1.5	89.5 ± 1.5	90.9 ± 1.2
	INV-PHP	78	84.6 ± 1.8	88.9 ± 1.6	91.5 ± 1.6	88.9 ± 2.0	96.6 ± 0.9	95.9 ± 0.9^d^	81.2 ± 2.4^f^	92.9 ± 1.6^f^	88.1 ± 1.6	88.1 ± 1.6^f^	90.0 ± 1.3
365	Control	81	86.1 ± 1.7	92.0 ± 1.4	93.8 ± 1.4	91.1 ± 1.8	97.0 ± 0.8	97.5 ± 1.0	86.4 ± 2.1	94.6 ± 1.3	90.4 ± 1.5	91.8 ± 1.3	92.2 ± 1.1
	INV-PHP	73	86.6 ± 1.8	92.5 ± 1.5	94.1 ± 1.5	90.8 ± 1.9	97.8 ± 0.9	95.6 ± 1.1	84.8 ± 2.2	94.7 ± 1.4	90.6 ± 1.5	90.7 ± 1.4	92.0 ± 1.2

^a^ *n* = 99, ^b^ *n* = 103, ^c^ *n* = 93, ^d^ *n* = 76, ^e^ *n* = 87, ^f^ *n* = 77

^g^Analysis of covariance (ANCOVA) with covariate Family Functioning Summary Score at Baseline (Day 14) was used to analyze all post-Baseline visits

*Statistically significant, p-value <0.05

**Table 15:** PedsQL Infant Scales - Acute scores at days 120, 180, 275, and 365

Age (Days)	Study Group	*n*	Physical Functioning		Physical Symptoms		Emotional Functioning		Social Functioning		Cognitive Functioning		Physical Functioning		Psychosocial Health Summary Score		Total Scale Score	
120	Control	86	91.3	(1.3)	88.6	(0.9)	87.7	(1.3)	95.6	(1.2)	93.8^e^	(1.4)	89.6	(0.9)	90.5	(1.0)	90.1	(0.9)
	INV-PHP	78	92.0	(1.4)	88.1	(1.0)	87.7^a^	(1.3)	96.3^c^	(1.3)	93.8^a^	(1.5)	89.6	(1.0)	90.6	(1.1)	90.2	(0.9)
180	Control	88	92.4	(1.3)	89.1	(1.1)	88.9	(1.3)	95.9	(1.0)	94.5	(1.1)	90.4	(1.1)	91.4	(1.0)	91.0	(1.0)
	INV-PHP	80	93.7	(1.4)	88.2	(1.1)	88.7	(1.4)	97.8	(1.0)	95.4	(1.2)	90.3	(1.1)	91.8	(1.1)	91.1	(1.0)
275	Control	85	95.4	(1.0)	94.6*	(0.9)	89.9^b^	(1.3)	96.8^d^	(0.8)	96.6^b^	(0.9)	94.9*	(0.8)	92.4	(1.0)	93.6	(0.8)
	INV-PHP	78	94.2	(1.1)	91.1	(0.9)	87.9	(1.3)	97.8	(0.9)	96.5	(1.0)	92.2	(0.9)	91.6	(1.1)	91.9	(0.9)
365	Control	81	96.1	(0.8)	94.5	(0.8)	91.4	(1.2)	96.5	(0.9)	97.3	(1.0)	95.1	(0.7)	93.6	(0.9)	94.3	(0.7)
	INV-PHP	73	96.9	(0.8)	94.0	(0.9)	90.5	(1.2)	97.9	(0.9)	96.7	(1.0)	95.1	(0.7)	93.2	(1.0)	94.0	(0.8)

*Statistically significant, p-value < 0.05

^a^ *n* = 77; ^b^ *n* = 84; ^c^ *n* = 76; ^d^ *n* = 84; ^e^ *n* = 85

## Discussion

In this multi-center, double blind, randomized controlled trial, equivalence in rate of weight gain from 14 to 120 days of age was demonstrated in healthy term infants who received an Investigational PHP infant formula (total protein: 2.3 g/100 kcal and modified lactose: approximately, but no less than 50% of total carbohydrate) or Control IP formula (total protein: 1.9 g/100 kcal and lactose: approximately 92% of total carbohydrate) from 14 to 365 days of age. Mean group differences from 14 to 120 days and the 90% CI were within the predefined equivalence of ± 3 g/day. Few significant group differences in body weight or length growth rates were detected in any measured age interval. There were no significant group differences observed for head circumference growth rates for any age interval. No significant group differences were detected for mean achieved weight, length, or head circumference at any time point assessed with the exception of significantly lower mean achieved length in the Control compared to INV-PHP at Days 180 and 275. However, this statistical difference is not clinically meaningful given that the mean achieved length remained at approximately the 50th percentile of the WHO growth standard at these same timepoints. Mean achieved weight remained between the 25th and 75th percentiles of the WHO growth standard through Day 180 for male and female infants in both groups and subsequently tracked between the 50th and 90th percentiles through day 365. Z-scores also appeared to fall within the range described as “normal” using WHO guidelines. Overall, results from the present study add to previous data that demonstrate PHP formulas are safe, well-tolerated, and support adequate growth compared to intact cow’s milk protein formula.

Differences in growth between males and female infants are typical and have been well-recognized for decades [27]. In the present study, for each sex, approximately equal number of infants were assigned to each study group at randomization and all achieved growth anthropometrics were appropriately plotted by sex as recommended by expert bodies such as the WHO and the US Centers for Disease Control. For growth rates, achieved anthropometrics, and z-scores, a sex-by-study group interaction was included in the final statistical model when it was significant and a post-hoc analysis was performed with each sex analyzed separately. Although post-hoc analyses were reported here, the study was not originally powered to detect group differences by sex and all growth indicators appear to fall within typical ranges for healthy, term infants.

Acceptance and tolerance were good. No differences were observed in overall study discontinuation, study formula discontinuation, parental-reported fussiness, gassiness, or spit-up. Few group differences were detected in parental-reported crying, sleep, or stool frequency or consistency, quality of life, and formula intake measures during the study period. However, these were either at isolated or nonconsecutive time points and therefore do not appear to be clinically meaningful. Both formulas in this study had the prebiotic blend of PDX: GOS. PDX: GOS added to intact cow’s milk protein infant formula has been previously demonstrated as well-tolerated, supported normal growth, and promoted softer stools in healthy term infants when compared to infants who received formula with no added prebiotic blend [15, 20, 28, 29], and yield stool consistency more closely to those of breast-fed infants [15]. No differences between study formula groups were detected in the number of participants who had at least one medically confirmed adverse event reported, nor for specific categories (allergic conditions and GI infections). Additionally, parental reports of gassiness, fussiness, spit-up, crying, sleep, and quality of life demonstrated similar and consistent measures of comfort across both groups, suggests the Investigational PHP formula maintains a favorable tolerance profile comparable to traditional intact cow’s milk protein formulas. Overall, this study supports that PHP formula with added PDX: GOS was well-tolerated, safe, and supports adequate growth in infants receiving formula.

Strengths of this study include the randomized, prospective, controlled design and study feeding period through one year of age. The rigorous statistical approach in this study, with an equivalence margin of ± 3 g/day for weight gain, further strengthens results reported here. The study design, with a sample size selected for an 80% power, provides robust evidence supporting the conclusion of equivalency. Also in accordance with AAP guidance [21], the Investigational PHP formula was compared to a previously marketed formula demonstrated to support adequate growth in infants receiving formula.

The current study was designed to compare two different formulas under controlled conditions; infants were enrolled only after caregivers independently decided to exclusively feed infant formula, consistent with guidance for growth and tolerance studies [30]. An additional breastfed reference group was not enrolled and may be considered a limitation. However, the WHO reference standards reflect growth trajectories of breastfed infants; therefore, plotting participant growth on the WHO growth curves was used as a pre-specified proxy for this reference group [31]. Another potential limitation is the subjective nature of some secondary outcomes via parent-reported questionnaires. However, parent-reports of outcomes such as crying, fussiness, and gassiness are clinically relevant, can affect caregiver and family well-being, and are not easily measured by objective means and thus were assessed using previously published parent-reported clinical outcomes [17, 20, 24, 32, 33] and where possible validated questionnaires, such as the PedsQL [25]. Furthermore, randomization ensures groups can be compared. Finally, PDX: GOS has been found to support stool microbiota composition closer to that of breastfed infants [15], but because a breastfed reference group was not enrolled and no stool samples were collected during this trial, other potentially relevant outcomes such as microbiota composition were not assessed. In addition, future clinical outcomes past 12 months of age were not in scope of this clinical trial. These remain potential topics for future studies.

## Conclusions

A partially hydrolyzed cow’s milk protein infant formula with an added prebiotic blend of PDX: GOS was associated with age-appropriate growth through one year of age in healthy, term infants. For infants who received a Control IP formula or the Investigational PHP formula, stools remained soft, in line with previous data reported for addition of PDX: GOS to infant formulas. By aiming to model functional outcomes of human milk feeding (e.g., stool characteristics through addition of prebiotics), formulas may support overall infant health and ease of feeding, particularly as advancements continue in formula composition and gastrointestinal health research. Overall, the study demonstrated that partially hydrolyzed cow’s milk protein infant formula with an added prebiotic was safe, well-tolerated, and associated with adequate growth for healthy term infants receiving formula through one year of age.

## Data Availability

The authors and study sponsor encourage and support the responsible and ethical sharing of data from clinical trials. De-identified participant data from the final research dataset used in the published manuscript may only be shared under the terms of a Data Use Agreement. Requests may be directed to: veronica.fabrizio@reckitt.com.

## Abbreviations

AAP: American Academy of Pediatrics
GOS: Galactooligosaccharides
PDX: Polydextose
PedsQL: Pediatric Quality of Life
PHP: Partially hydrolyzed cow’s milk protein
WHO: World Health Organization
